# High-Yield and High-Accuracy Mass Transfer of Full-Color Micro-LEDs Using a Blister-Type Dynamic Release Polymer

**DOI:** 10.1021/acsami.5c01531

**Published:** 2025-04-29

**Authors:** Xinrui Huang, Qian Liu, Jinkun Jiang, Xuehuang Tang, Xin Lin, Yujie Xie, Taifu Lang, Zhonghang Huang, Qun Yan, Chang Lin, Jie Sun

**Affiliations:** † National and Local United Engineering Laboratory of Flat Panel Display Technology, College of Physics and Information Engineering, Fuzhou University, Fuzhou 350100, China; ‡ Fujian Science & Technology Innovation Laboratory for Optoelectronic Information of China, Fuzhou 350100, China; § Rich Sense Electronics Technology Co., Ltd., Quanzhou 362200, China; ∥ Quantum Device Physics Laboratory, Department of Microtechnology and Nanoscience, Chalmers University of Technology, Gothenburg 41296, Sweden

**Keywords:** micro-LED, mass transfer, laser, blister-type, full-color, DRL

## Abstract

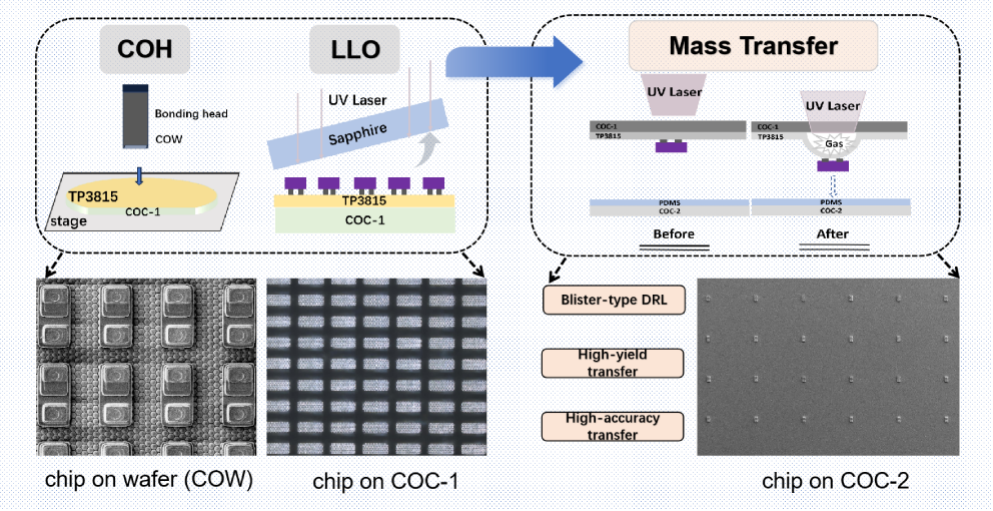

Micro light-emitting diode (Micro-LED) is widely regarded as a highly promising technology in the current display field due to its excellent performance, but the core issue hindering the further development of Micro-LED is how to achieve high-precision and high-yield transfer. In this study, laser-induced forward transfer (LIFT) is adopted as the main technique, and a novel blister-type dynamic release layer (DRL) material is selected, characterized by a gentle transfer process and minimal residue on the chip after transfer. Chip-on-wafer (COW) is a structure that fabricates a large number of Micro-LEDs (15 × 30 μm^2^) on a sapphire substrate. The COW-on-head (COH) chip bonding method can control the uniformity of the overall chip height before transfer within 3.5%, which is favorable for subsequent stable transfer. Based on the analysis of the close relationship between the transfer gap and laser energy density, this study successfully achieved the transfer of red/green/blue (R/G/B) Micro-LED chips (6400, respectively) onto the corresponding chip-on-carrier 2 (COC-2), and all of them have achieved a one-step transfer yield of over 99.3% and an average chip transfer offset of 2 μm or less. It is worth mentioning that the one-step transfer yield mentioned in this paper is different from the yield after testing and repairing the chips. The one-step transfer yield can fully reflect the transfer quality. In order to verify the validity of this study, a 1 in., full-color, active Micro-LED display with a pixel size of 114 pixels per inch (PPI) and a display brightness of 5598 cd/m^2^ was successfully fabricated. This study proposes an optimized solution for Micro-LED transfer technology, which will help accelerate the mass production and marketization of Micro-LED.

## 1 Introduction

Micro-LED displays consist of arrays of light-emitting diodes (LEDs) on the micrometer scale. Typically, an LED with a mesa size of less than 50 μm is defined as a Micro-LED,^1^ which offers higher brightness, faster response time, lower energy consumption, and longer service life^2,3^ compared to traditional liquid crystal displays (LCDs)^4,5^ and organic light-emitting diodes (OLEDs).^6,7^ As a result, it has garnered significant attention from the display industry in recent years.^8^ Micro-LED displays are currently demonstrating substantial application potential in various devices, including smartwatches,^9^ virtual reality (VR), and augmented reality (AR)^10^ systems.

Although Micro-LEDs have broad development prospects, they are also facing serious technical bottlenecks. It is more difficult to achieve the direct integration of Micro-LEDs with driver substrates using existing material growth processes, making the transfer process particularly important for establishing the electrical connection between chip arrays and driver substrates.^11^ The mass transfer process involves separating a large number of Micro-LED chips from the growth substrate and placing them precisely on the target substrate.^12^ However, because the Micro-LED chip size is exceedingly small and the total number of transferred chips is immense, achieving efficient, high-precision, and high-yield transfer has become a major challenge. This also hinders the further development of Micro-LEDs toward large-scale industrialization. To address the existing challenges of Micro-LED mass transfer, current technologies are developing rapidly, including electrostatic transfer,^13^ electromagnetic adsorption transfer,^13^ stamp transfer,^14,15^ laser-induced forward transfer (LIFT),^16,17^ and others. Among these, electrostatic transfer can transfer a large number of chips in a single pass but may cause chip rupture. Electromagnetic adsorption transfer can flexibly adjust the magnetic force, but its accuracy is significantly affected by the uniformity of the magnetic materials. In recent years, stamp transfer and LIFT have emerged as two mainstream processes for achieving Micro-LED mass transfer. Stamp transfer is characterized by high precision and reusability,^18^ but its relatively low transfer efficiency is a key factor limiting its further development.^19^ In contrast, LIFT stands out for its significant advantages. The core principle involves spin-coating a transparent substrate with a dynamic release layer (DRL) that absorbs a specific range of laser wavelengths. The energy from the laser beam penetrates the transparent substrate and is absorbed by the DRL, causing thermal expansion at the interface and resulting in the melting or vaporization of the DRL. This process causes the chip to detach from the transparent substrate and transfer to a temporary substrate,^20^ often referred to as a carrier. Recently, a study demonstrated the transfer of more than 100 M Micro-LEDs per hour by using laser mass parallel transfer technology,^21^ highlighting the strong advantage of LIFT in achieving high-efficiency transfer of micron-sized chips. So far, LIFT has shown great potential in the field of Micro-LED mass transfer.

In LIFT, the blister-type DRL is one of the main types of DRL.^22^ The principle is that the blister-type DRL reacts with the laser to form a gas bubble that reduces the contact area between the DRL and the chip, thereby promoting the chip’s transfer.^23^ A high-quality blister-type DRL material should leave minimal residue on the surface of the chip after transfer, ensure a gentle transfer process, and enable high-precision and high-yield transfer. However, there are increasingly fewer mature blister-type materials that meet these requirements. Fardel et al.^24^ transferred a chip using photolyzed triazene polymers, which reduced the residual DRL material on the device surface. However, during small-distance transfers, the reflected shock wave could damage the chip. Conversely, in long-distance transfers, the chip disintegrated before reaching the target substrate. Min et al.^25^ used a homemade photoresist (PR) as a DRL to produce liquid carboxylic acid under UV irradiation. Upon heating, the liquid carboxylic acid vaporizes, causing the PR to expand and push the chip for transfer. Although this study achieved a chip transfer accuracy within ± 1.2 μm, it required strict control of the heating process to ensure the carboxylic acid vaporized evenly. Uneven heating could lead to irregular expansion of the PR, thereby affecting the uniform release of the chip and transfer yield.

To address the existing problems, in this study, we carefully selected a customized new blister-type DRL called TP3815 (Toray Industries, Inc.), which is capable of being applied to Micro-LED chips. Compared with some blister-type materials, it has the characteristics of less residue on the chip surface after transfer, a gentle transfer process, and no extra heating treatment during the transfer. Chip-on-wafer (COW) is a structure that includes a large number of Micro-LEDs on a sapphire substrate. In this work, we have used a COW-on-head (COH) method for chip bonding, which ensures that the uniformity of the overall chip height is less than 3.5% before the transfer, laying the foundation for subsequent transfer with high yield and high precision. Then, we analyzed the close relationship between the transfer gap and the laser energy density, on the basis of which we successfully transferred red/green/blue (R/G/B) Micro-LED chip arrays (6400 each) onto chip-on-carrier 2s (COC-2s) using a 266 nm UV laser. For all of them, we have achieved high one-step transfer yields of over 99.3% and average transfer offsets of 2 μm or less per chip. It is worth mentioning that the one-step transfer yield mentioned in this paper refers to the initial yield of the chips transferred to COC-2, which is different from the yield after repair. The one-step transfer yield fully reflects transfer quality. Finally, a 1-in. R/G/B active Micro-LED display with a pixel density of 114 pixels per inch (PPI)^26^ and a pixel pitch of 222 μm was successfully prepared by bonding the COC-2s to the thin-film transistor (TFT)^27^ driver substrate. The brightness of the display reached 5598 cd/m^2^. We believe that this study serves as an important reference for further promoting the realization of high-yield and high-precision transfer of Micro-LEDs.

## 2 Experiment

### 2.1 Bonding Micro-LED Chips to COC-1

**Figure 1:**
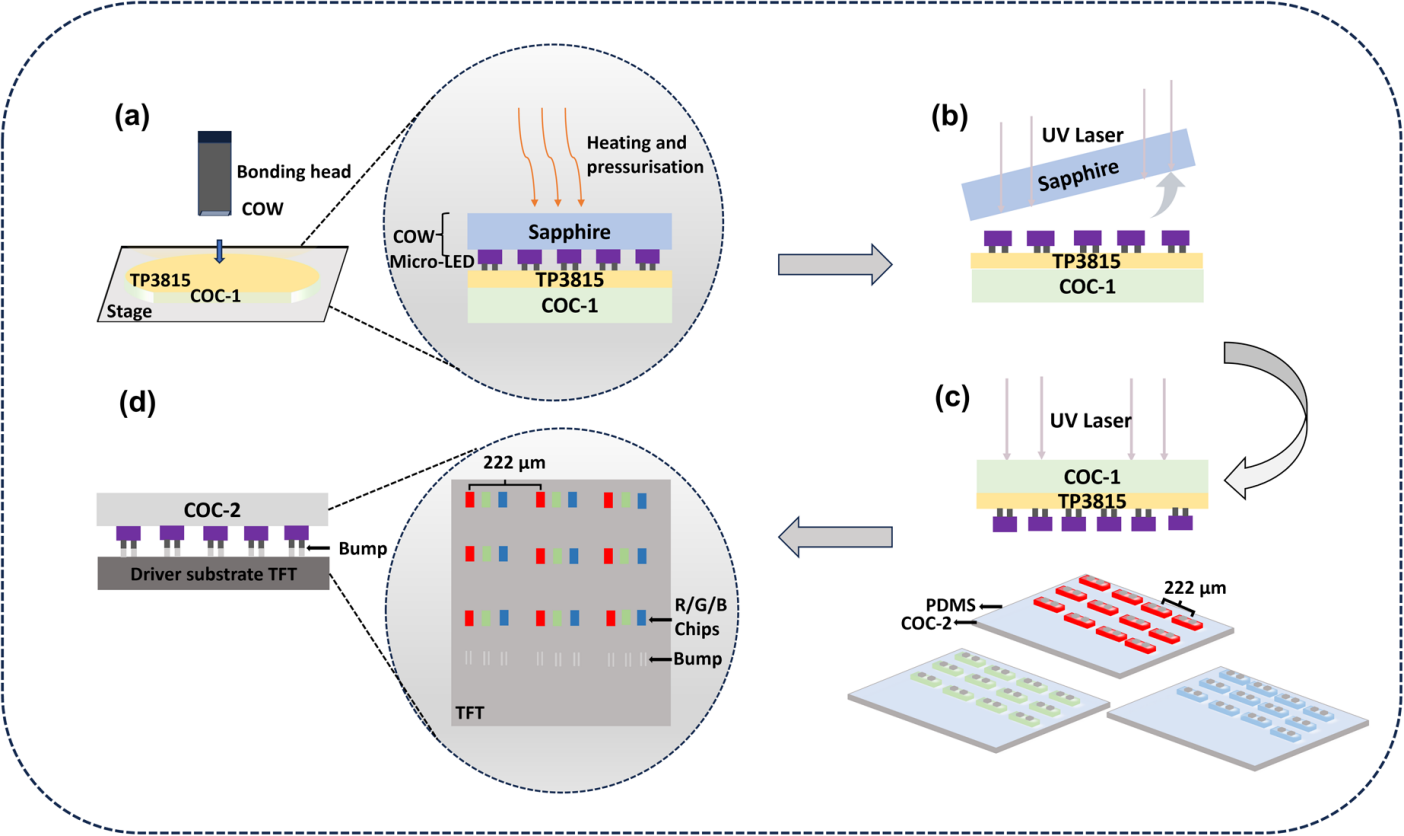
Overall steps of the experiment. (a) Bonding the Micro-LED chips on a COW to a COC-1. (b) LLO. (c) Transferring the Micro-LED chips on COC-1 to COC-2 by laser. (d) Bonding the chips on COC-2 to the bumps on the TFT driver substrate.

First, the DRL material TP3815 with a thickness of 15 μm is spin-coated onto the chip-on-carrier 1 (COC-1) made of quartz and baked for 5 min at 120 °C on a hot plate. Subsequently, it is placed in an oven and heated at 255 °C for 30 min for postbake curing. COW is sequentially cleaned with acetone, isopropanol, and deionized water and blown dry with nitrogen. COH indicates that COW is attached to the bonding head of the multifunctional flip-chip bonding machine, while COC-1 is adsorbed onto the stage of the bonding machine by vacuum. When the bonding head and the stage are heated to 70 and 30 °C, respectively, a pressure of 0.5 MPa is applied and maintained for 5 min to achieve the bonding of COW to COC-1. The entire process is shown in Figure 1a.

### 2.2 Stripping of Sapphire Substrate on COW

In order to achieve the patterned transfer of chips, the stripping of sapphire by a laser is a necessary and critical part of the process. This process is called laser liftoff (LLO)^28^ as shown in Figure 1b. The UV laser rapidly passes through the sapphire substrate on the COW, scanning and heating the interface between the epitaxial layer and the sapphire substrate. Taking blue/green Micro-LEDs (see Section S1, Section S2, Figures S1 and S2 for more details about R/G/B Micro-LEDs) as an example, GaN as the epitaxial layer is partially decomposed by heat to generate nitrogen and gallium metal^29^ (eq 1), which weakens the bonding force between the materials. Thus, the sapphire substrate is stripped off.
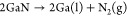
1

### 2.3 Transferring Micro-LED Chips to COC-2

After the LLO, the electrodes of the Micro-LED chip are now embedded in TP3815 on the surface of COC-1. However, in order to make the chip electrodes face outward so that they can be bonded with the bumps on the TFT driver substrate to realize the light-up, we need to transfer the Micro-LED chips to COC-2 by laser, as shown in Figure 1c. The pitch between the centers of adjacent chips after transfer is 222 μm. COC-2 is made of 3 cm × 3 cm ITO (Indium Tin Oxide) glass and spin-coated with viscoelastic polydimethylsiloxane (PDMS)^30^ for the reception of the Micro-LED chips. This process uses a 266 nm UV laser. This wavelength of the laser passes through COC-1 and is absorbed by the DRL material TP3815 to create a bubble effect. The chip is given enough momentum to be transferred efficiently. In our experiments, the transfer time for a single Micro-LED chip is 0.6 s, or about 1.67 chips per second. In the end, a one-step transfer yield of 99.3% was achieved for 6400 chips, with an average transfer offset of 2 μm or less per chip.

### 2.4 Bonding

In the final step, as shown in Figure 1d, the COC-2s containing the R/G/B color chips are successively bonded to the bumps on the TFT driver substrate. The pitch of neighboring bumps for the same color is 222 μm, and this process uses micron-scale bonding equipment that can accurately bond the chips to the corresponding positions on the TFT driver substrate.

## 3 Results and Discussion

### 3.1 Uniformity of Micro-LED Chips Bonding to COC-1

**Figure 2:**
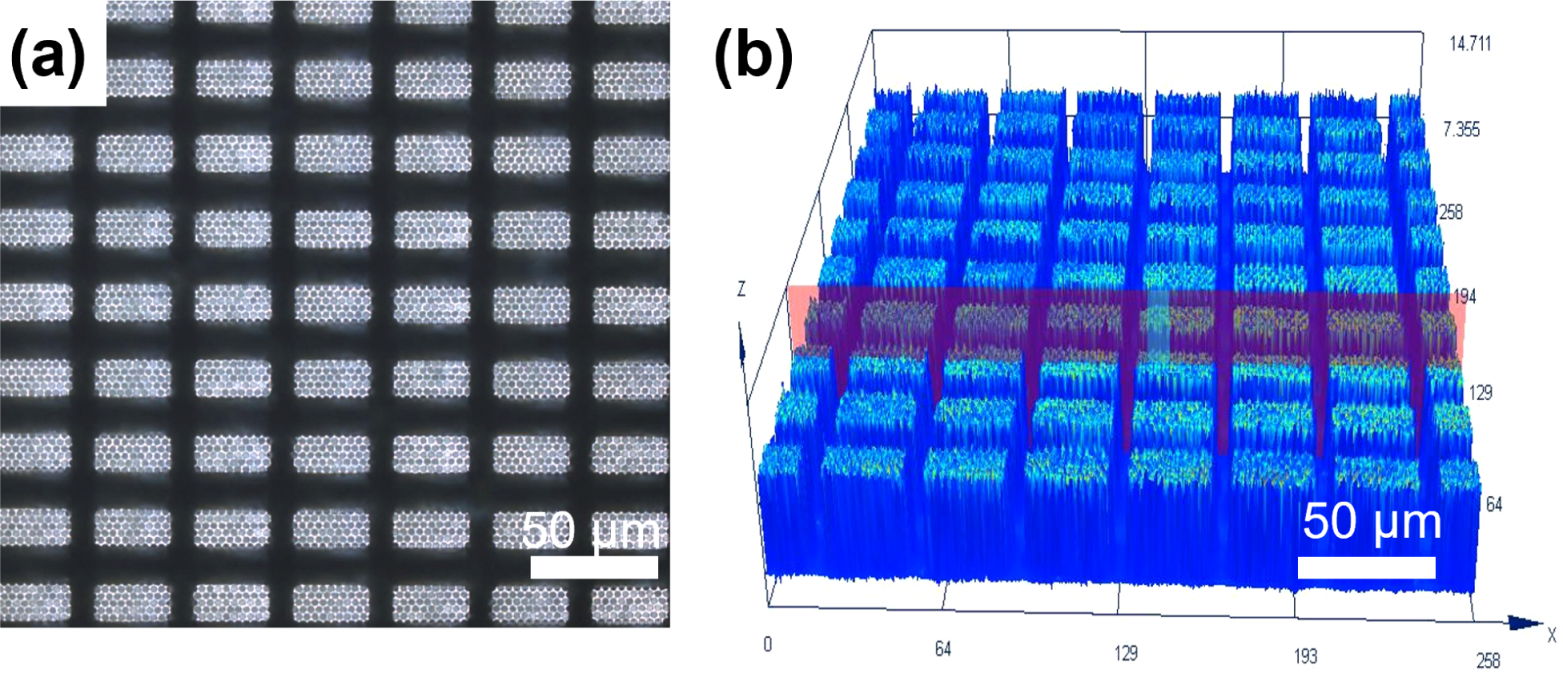
(a) Image of the chip array after the LLO under a microscope at 50×. (b) 3D image measuring the height of the TP3815 adhesive surface to the top of the chip.

The uniformity of the bonding of the Micro-LED chips to COC-1 is an important factor that affects the subsequent transfer yield. Uneven bonding means that chips in different areas on the COW will have different depths at which they are embedded in TP3815, leading to inconsistent and unstable results when the same laser energy density is used for the subsequent transfer. As shown in Figure 1a, we adopt the bonding method of adsorbing COC-1 on the stage and the bonding head adsorbing the COW for downward bonding. The bonding head is made of alumina ceramic, which has a Mohs hardness of 9. The high hardness means that the COW is not easy to deform under pressure,^31^ which helps to alleviate the warping of the COW during bonding and improves the uniformity of the depth of the chip sink. Since the height of the TP3815 adhesive surface to the top of the chip can reflect the depth of the chip embedded into the TP3815 to a certain extent (Figure 2a,b), the height of the chips in the five regions after bonding the R/G/B COWs to COC-1 and stripping the sapphire was measured using a three-dimensional laser microscope. The specific data are presented in Table 1. The overall chip height uniformity was calculated using eq 2. A smaller uniformity value indicates a smaller chip height difference. The calculated chip height uniformity for R/G/B Micro-LED chip arrays on COC-1 is 1.373%, 3.156%, and 2.276%, respectively. This demonstrates that COH results in excellent chip height uniformity, which lays a solid foundation for the subsequent improvement of transfer precision and yield.
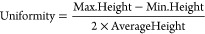
2

**Table 1:** Chip Heights in the Five Regions after the LLO

	Upper left (μm)	Upper right (μm)	Mid (μm)	Lower left (μm)	Lower right (μm)	Average height (μm)	Uniformity
Red	8.392	8.474	8.626	8.572	8.547	8.522	1.373%
Green	6.745	6.816	6.970	6.542	6.831	6.781	3.156%
Blue	6.814	6.619	6.927	6.672	6.793	6.765	2.276%

### 3.2 Gentle Transfer Process

**Figure 3:**
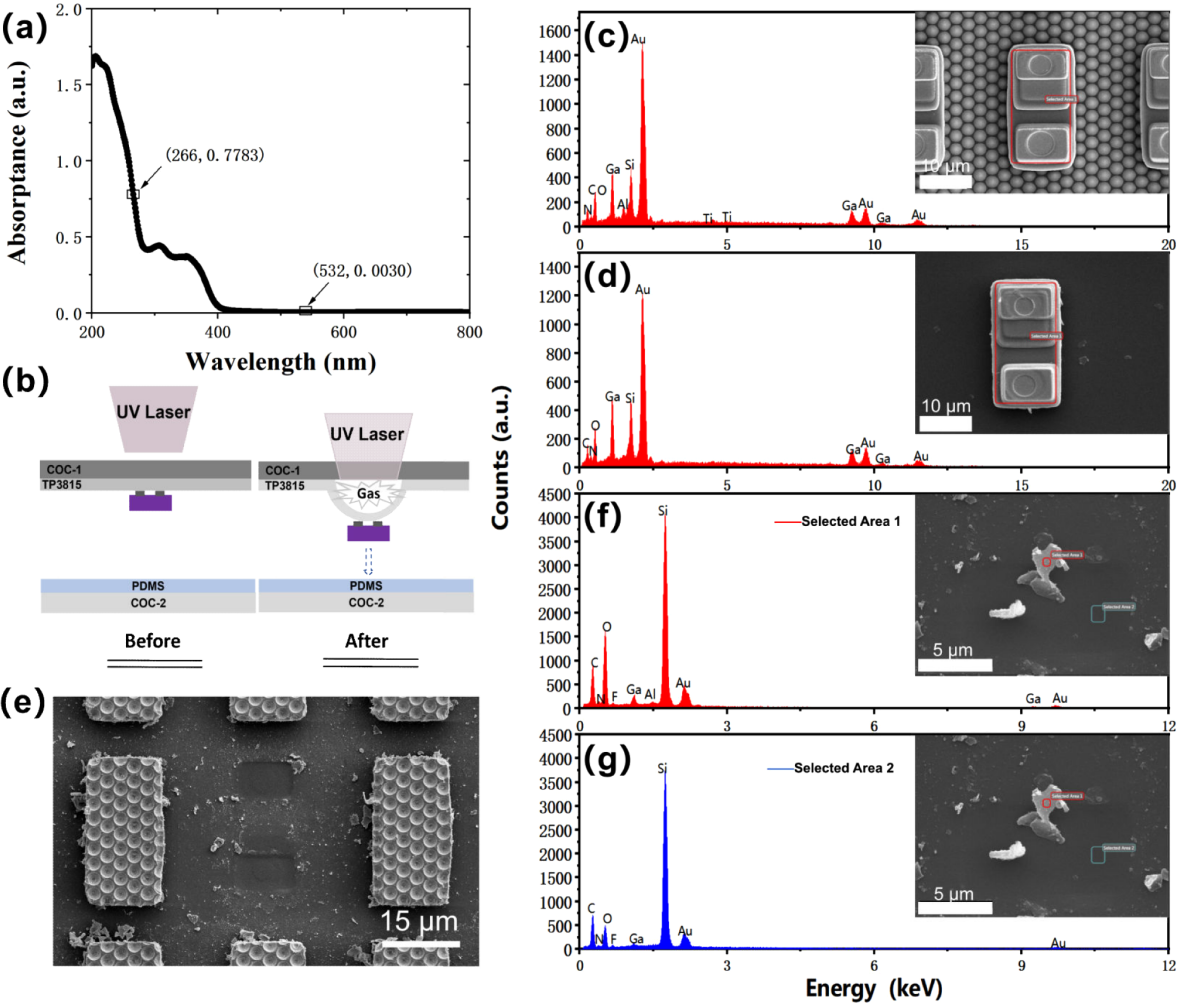
(a) Absorption spectrum of TP3815. (b) Schematic diagram comparing the state of TP3815 before and after being irradiated by the laser. (c) EDS analysis of the chip on the COW. (d) EDS analysis of the chip on COC-2. (e) SEM image of COC-1 after transferring. (f) EDS analysis of the debris on COC-1 after transferring. (g) EDS analysis of the surface of TP3815 after transferring.

The laser transfer device used in this study, LMT-350TRF (Toray Engineering Co., Ltd.), provides two laser wavelengths, 266 and 532 nm, respectively. Figure 3a shows the absorbance of the TP3815 with a film thickness of approximately 303 nm for lasers with wavelengths ranging from 200 to 800 nm measured using Cary-7000. It was found that the absorbance is only 0.0030 au for a laser with a wavelength of 532 nm, while it reaches 0.7783 au for a wavelength of 266 nm. What is more, the short wavelength of the 266 nm UV laser means that the photon energy of the laser is higher, which can provide finer processing capability.^32^ Therefore, we chose a 266 nm UV laser to transfer the chip.

Figure 3b depicts a schematic diagram comparing the state of TP3815 before and after being irradiated with the laser. Before the transfer, TP3815 provides enough adhesive force to bond with the chip. After laser irradiation, the laser passes through COC-1 and acts on TP3815, which absorbs energy and ablates to produce gas. While reducing adhesion with the chip, it provides kinetic energy to the chip to facilitate its transfer.^23,33^

However, if the residue of the DRL material on the chip after the transfer is high, it will affect the electrical and mechanical connections between the chip and the TFT driver substrate during the subsequent bonding process. In order to determine the residue of TP3815 on the Micro-LED chip after transfer, a laser with a wavelength of 266 nm and an energy density of 0.415 J/cm^2^ was used to transfer the blue Micro-LED. We performed energy-dispersive spectrometer (EDS) analysis on the chip on COW (Figure 3c) and the chip transferred to COC-2 (Figure 3d), respectively. No new components appeared on the chip surface, nor was the content of any specific component significantly elevated after the transfer, indicating that there was very little TP3815 residue on the chip surface. Figure 3e shows the image obtained by observing COC-1 by scanning electron microscopy (SEM) after the transfer. Similarly, the debris composition in the image and the composition on the surface of TP3815 were analyzed by EDS (Figure 3f,g). After comparison, the main components of the debris were Ga and Ga oxides, which is due to the fact that GaN devices are prone to leaving some residues at the interfaces after being decomposed by laser irradiation, and these residues can be cleaned up by diluted hydrochloric acid.^34^ Combined with the SEM images and the above analysis, TP3815 did not experience bubble rupture during the process of vaporization by laser irradiation or during the process of transferring the chip, so there was almost no gas or other incompletely vaporized materials left on the chip surface. At the same time, the laser with an energy density of 0.415 J/cm^2^ is also one of the factors affecting chip transfer. The appropriate laser intensity can control the bubble in a state that will not rupture and is easy to transfer the chip, thus enabling a gentle chip transfer.

### 3.3 The Effect of the Laser Energy Density and the Transfer Gap on Transfer

**Figure 4:**
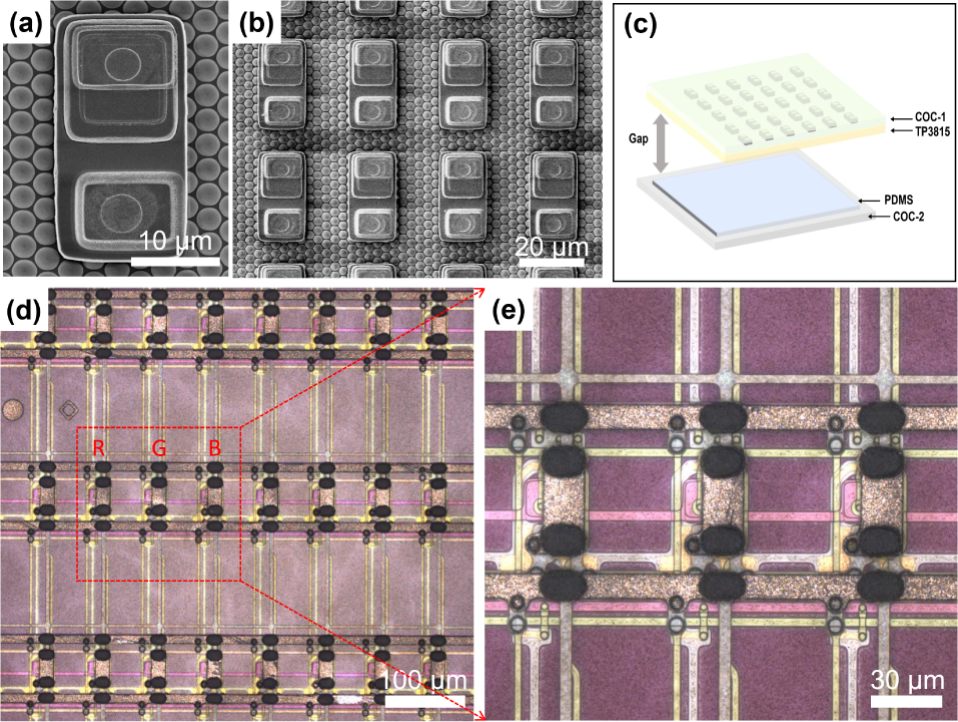
(a) SEM image of a Micro-LED chip. (b) SEM image of a part of the Micro-LED chip array on a COW. (c) Schematic of the gap. (d, e) Image of a TFT bump array under a microscope.

For the process of transferring the chips onto COC-2, achieving a one-step transfer with high precision and high yield is the goal to be accomplished. The SEM images of a single Micro-LED chip and a part of the chip array on a COW are shown in Figure 4a,b. The size of the chip is 15 × 30 μm^2^, so we determined the spot size to be 30 × 38 μm^2^ to ensure uniform irradiation of the entire chip. The pitch between the centers of the adjacent chips on the COW is approximately 27.5 μm in the *x*-direction and 37 μm in the *y*-direction. As shown in Figure 4c, the transfer gap refers to the flight distance at which the chip is dislodged from TP3815 to COC- 2.^23^

**Figure 5:**
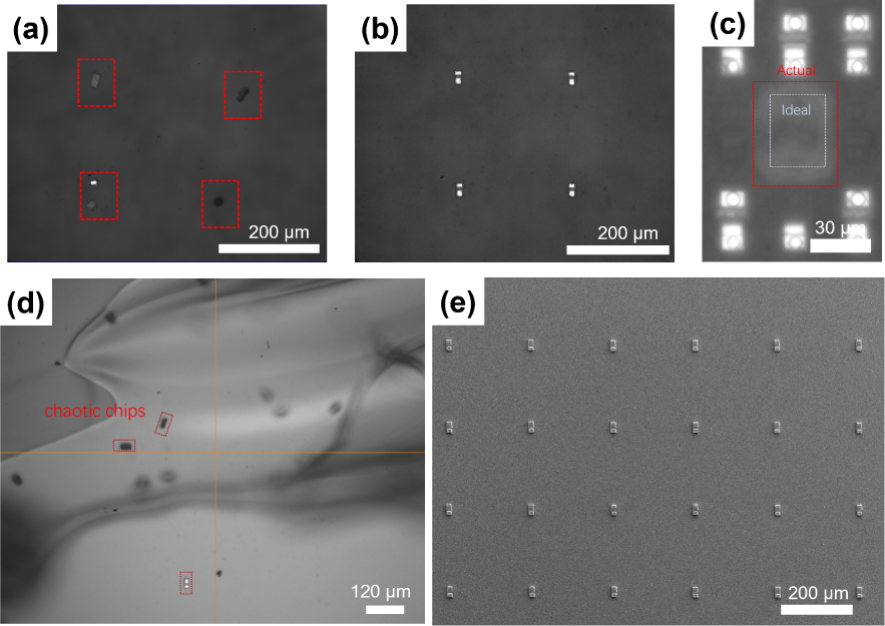
(a) Image of the flipped chips and the broken chip. (b) Image of successful chip transfer. (c) Image of actual spot size vs set spot size at laser energy density of 0.626 J/cm^2^. (d) Image of scratching at a gap of 20 μm. (e) Image of a part of the chip array on COC-2.

The array of photosensitive conductive polymer^35^ bumps on the TFT driver substrate is shown in Figure 4d,e. The size of a single bump is 20 × 12 μm^2^. To ensure the accuracy of the alignment of the chips when they are bonded to the bumps on the TFT driver substrate as much as possible, we define a chip with an offset of more than 5 μm in the *x*- and *y*-directions after transfer as a bad chip. In addition to this, bad chips also include broken, missing, and flipped chips, as shown in Figure 5a.

Table 2 lists the correspondence between the laser energy ratio and the laser energy density. We found that when the laser energy ratio was less than or equal to 42%, the chip would not be able to detach from TP3815. This may be due to the fact that the smaller laser energy density results in less expansion of the bubble, making it difficult to debond the chip, and the kinetic energy generated in the transient is not sufficient to drive the chip to transfer. When the laser energy ratio was increased to 46%, the chip could successfully fall onto COC-2 at a gap of 25 μm, but when the gap continued to be increased to 30 or 40 μm, the chip would always exhibit a flipped state. This suggests that if the gap is increased to a certain level at the same laser energy density, then the chip will flip during the drop. This may be due to the fact that the initial kinetic energy of the chip is small when the laser energy ratio is low; if the gap is too large, the chip will continue to lose kinetic energy to a certain extent when falling, and the surrounding environmental factors will start to play an influential role in causing the chip to flip. When the laser energy ratio was greater than 50%, even though the falling process was long (30–40 μm), the kinetic energy was large enough to overcome the influence of environmental factors, and the chips were able to fall accurately onto COC-2 (Figure 5b). Therefore, the gap and laser energy density have a close connection with mutual constraints. However, higher laser energy is not necessarily better, as shown in Figure 5c. When the laser energy ratio was greater than 60%, the bubble expansion was larger, and the actual spot size would be much larger than the set value, even affecting the surrounding chips. Similarly, a smaller gap is not always better. We tried a gap of 20 μm, but due to the close proximity of the upper and lower substrates, there was a risk of scratching. Figure 5d shows an image of the PDMS adhesive surface on the COC-2 being lifted up and the chip array being disorganized after the scratch. After several attempts, we determined to control the gap at 30–40 μm and the laser energy density at 0.315–0.495 J/cm^2^. If the parameters are adjusted according to the actual situation within the appropriate range of gap and laser energy density before each transfer, the one-step transfer yields of the R/G/B chip arrays onto COC-2s can reach over 99.3%, and the transfer accuracy of a single chip is within 2 μm on average. Figure 5e shows a part of the chip array on COC-2 after the transfer, and the rest of the array is almost the same except for some bad chips.

**Table 2:** Correspondence between the Laser Energy Ratio and Laser Energy Density

Energy ratio	42%	46%	50%	60%
Energy density (J/cm^2^)	0.182	0.248	0.315	0.626

### 3.4 The Transfer Accuracy of Chip Arrays

**Figure 6:**
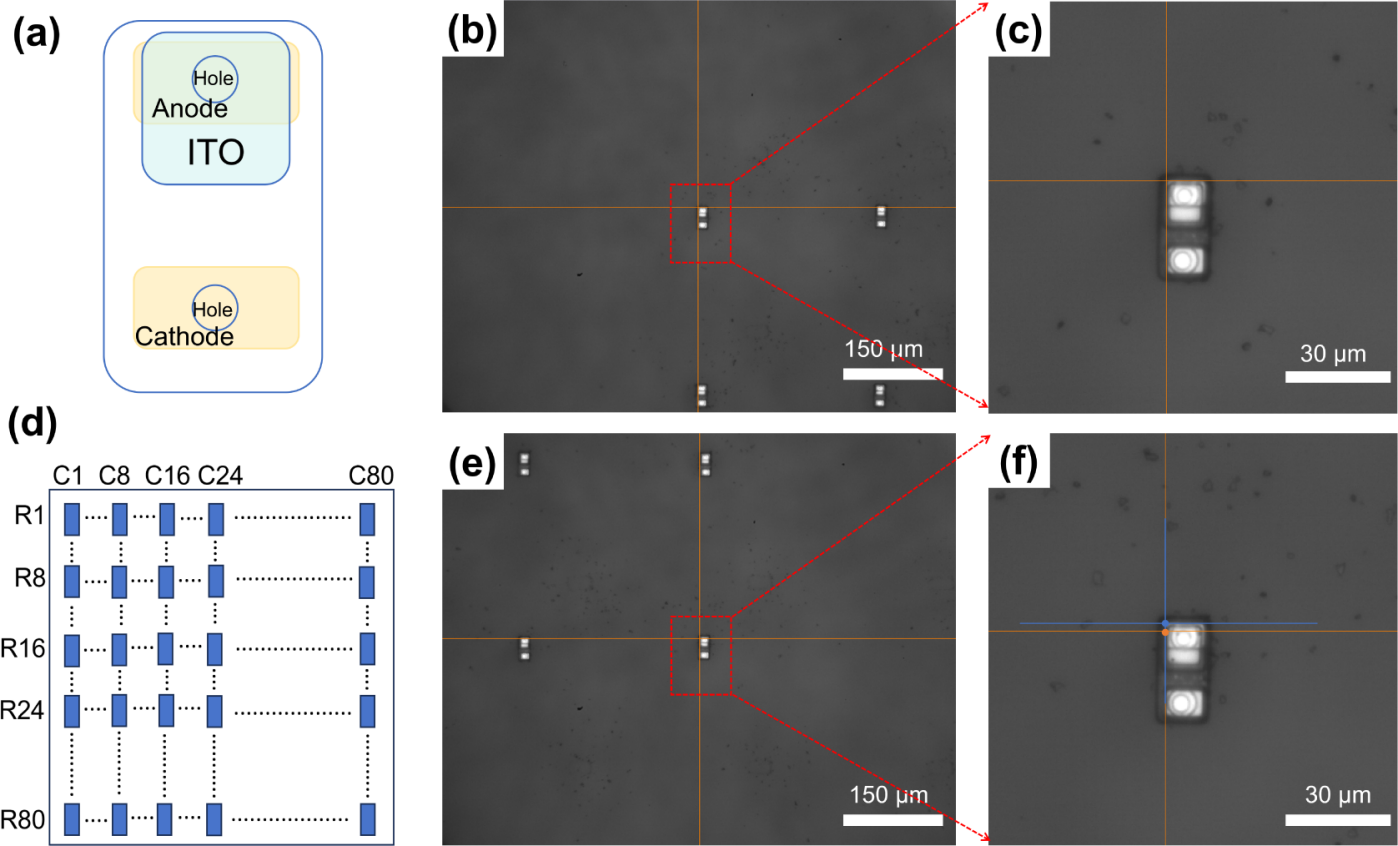
(a) Schematic of the structure of the Micro-LED chip. (b,c) Image of the first chip in an 80 × 80 chip array. (d) Schematic representation of transfer accuracy of statistical chip array. (e,f) Image of the chip under test.

**Figure 7:**
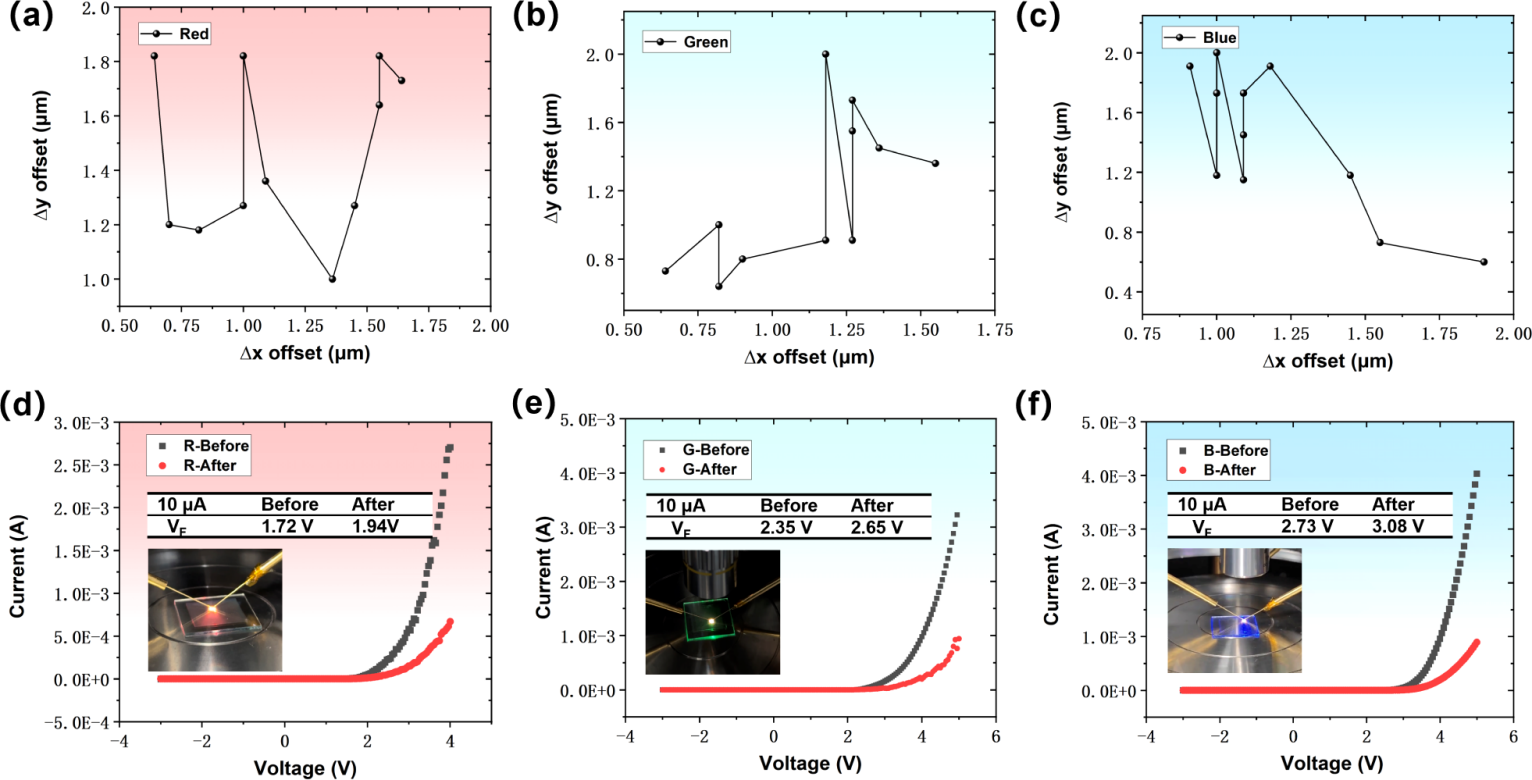
(a–c) Average transfer offset of the R/G/B chip arrays. (d–f) I–V characteristics of R/G/B Micro-LEDs before LLO and after transfer.

The structure of the Micro-LED chip is shown in Figure 6a. Since the cathode and anode electrodes of the chip have a more obvious difference from other areas, the upper-left corner of the anode of the first chip in the 80 × 80 chip array is defined as the origin (Figure 6b,c). The laser transfer device has the functions of distance measurement and moving at a fixed pitch. The yellow solid line in Figure 6e,f represents the ideal position of the upper-left corner of the anode of the chip under test, while the blue solid line represents the actual position. As shown in Figure 6d, this study measured one set of data every 8 rows, starting from the first row. Each set of data consists of the offsets Δ*x*_*i*_ and Δ*y*_*i*_ of the actual position of the upper-left corner of the chip anode from the ideal position in the *x*- and *y*-directions, which are measured every 8 columns starting from the first chip in this row. A total of 11 sets of data are obtained from rows 1 to 80, and Figures 7a–c show the offset values Δ*x* and Δ*y* after summing and averaging each set of data, which are calculated as in eq 3. This statistical method is able to take into account the overall accuracy of the chip array to a certain extent. It can be seen that the R/G/B three-color chip arrays are able to achieve an average transfer offset of a single chip within 2 μm. Accurate chip transfer can reduce the electrical connection problems caused by chip misalignment during subsequent bonding. In order to verify whether the transfer process has an effect on the optoelectronic characteristics of the chips, we measured the I–V characteristics of the R/G/B chips before the LLO and after transfer to COC-2 (Figures 7d–f). The results show some degree of forward current attenuation and an increase in the forward voltage (V_F_) at the same current (10 μA) after the transfer for the three colors of chips. Compared with reported works,^37,38^ the difference in the I–V characteristics before and after the transfer in this work is more evident. This may be due to unintentional thermal damage to the active region as well as other epilayers caused by the heat generated when the laser irradiates the GaN and the DRL during LLO and transfer, respectively. The increase in defect density may, however, increase the series resistance of the device, leading to an increase in the V_F_ at the same current. In order to improve this situation, optimizing the UV laser irradiation conditions, such as laser pulse frequency, laser energy density, etc., is the main research direction of our future work.
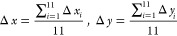
3

### 3.5 Flip-Chip Bonding and R/G/B Active Micro-LED Display Performance

**Figure 8:**
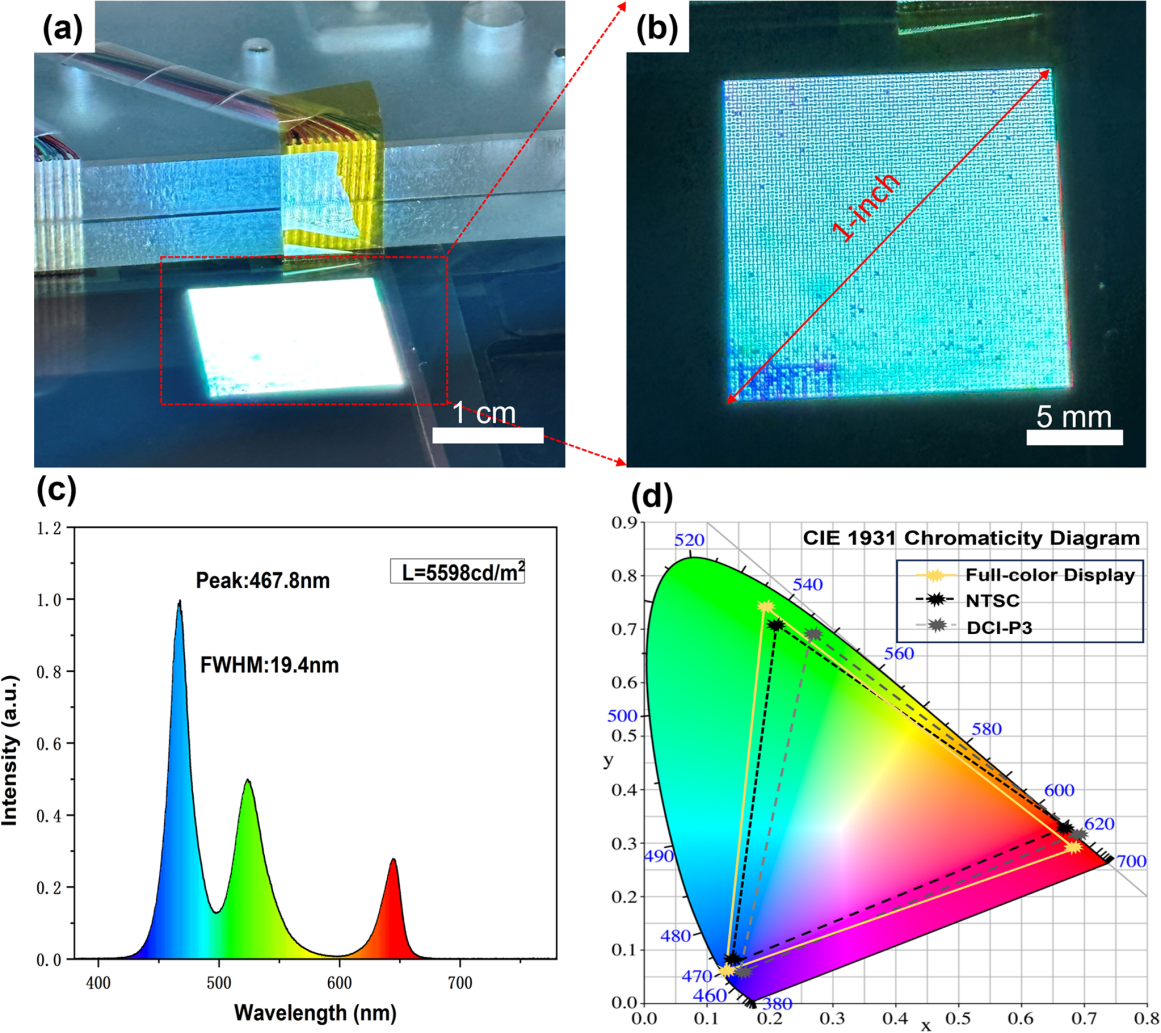
(a,b) Micro-LED display after bonding R/G/B chip arrays. (c) EL spectrogram of the Micro-LED display. (d) Color gamut areas of full-color Micro-LED display, NTSC, and DCI-P3, respectively.

In order to evaluate the luminescence performance of the transferred chip, it is first necessary to integrate the Micro-LEDs with a TFT driver substrate with bumps by means of a thermal compression bonding technique. This process is accomplished by applying a pressure of 27 MPa and a temperature of 180 °C for 5 min. The power delivery is performed through a field-programmable gate array (FPGA).^36^ The large-area lighting of a full-color Micro-LED display was successfully achieved and is shown in Figure S3. Our 1 in. full-color Micro-LED display currently achieves a yield of more than 90% (Figure 8a,b). The yield loss could be attributed to damaged chips during the transfer and potential alignment errors during bonding that affect electrical connection formation.

The electroluminescence (EL) spectrum of this Micro-LED display was obtained using an SRC-200M, as shown in Figure 8c. The brightness of the display reaches 5598 cd/m^2^. What is more, Figure 8d shows the color gamut area in CIE 1931 of the full-color Micro-LED display, which achieves 114.6% coverage of the NTSC standard and 119.3% coverage of the DCI-P3 standard (see Table S1 for more details about specific calculations). This indicates that the manufactured Micro-LED display is capable of displaying richer colors and covering a wider color gamut range, making it suitable for applications with higher color requirements.

Table 3 shows a comparison of several existing Micro-LED transfer studies in terms of transfer performance, the number of transferred chips, etc. It can be observed that the laser transfer method using a blister-type DRL material has certain advantages in terms of accuracy and yield when transferring microscale chips on a larger scale. On this basis, the full colorization of the display was realized. However, there is still some room for improvement in chip utilization, because the bubble generated by the blister-type DRL material after being irradiated by the laser may affect the surrounding chips. While this issue appears to be intrinsic, we are actively exploring methods to minimize the effect of bubbles on adjacent chips.

**Table 3:** Comparison of Several Existing Micro-LED Transfer Studies

Main transfer material	Chip size (μm^2^)	Number of chips transferred (pcs)	Transfer pitch (μm)	One-step transfer yield	Accuracy (μm)	Full-color display	Chip utilization	ref.
**Photoresist**	**15 × 30**	**–**	**75 × 75**	**–**	**1.2**	**No**	**High**	(25)
**Stamp**	**285 × 285**	**10 × 10**	**885 × 885**	**–**	**–**	**No**	**High**	(37)
**Tape**	**∼45 × 70**	**Wafer**	**–**	**∼99.8%**	**0.5**	**No**	**High**	(38)
**Tunable adhesive**	**280 × 280**	**<50**	**–**	**–**	**–**	**No**	**High**	(39)
**Blister-type DRL**	**15 × 30**	**80 × 80**	**222 × 222**	**≥99.3%**	**2**	**Yes**	**Medium**	**This work**

## 4 Conclusion

In this study, we utilized the LIFT technique as the main scheme for the transfer of Micro-LEDs (15 × 30 μm^2^) and used a novel blister-type DRL, which is characterized by less residue after the transfer and a gentle transfer process. The adopted COH chip bonding method ensures that the uniformity of the overall chip height is less than 3.5% before transfer. By analyzing the close relationship between the gap and laser energy density, we determined the appropriate ranges of gap and laser energy density, based on which we successfully achieved one-step transfer yields of 99.3% or more for transferring 80 × 80 R/G/B Micro-LED chip arrays to COC-2s, with the average transfer offset of the chips being within 2 μm. This study not only improves the transfer accuracy and yield but also realizes the large-area lighting of a 1 in. R/G/B active Micro-LED display, providing important reference suggestions for the process of mass transfer of Micro-LED chips, especially for further mass production and commercialization of Micro-LED displays.

## Supporting Information Available

Chromaticity coordinates of the Micro-LED display as well as calculation of color gamut area and coverage; the structures of R/G/B Micro-LEDs; optoelectronic characteristics of the R/G/B Micro-LEDs on COWs; the large-area lighting of a full-color Micro-LED display with red, green, and blue (PDF)
