# Supplementary material for: High-Yield and High-Accuracy Mass Transfer of Full-Color Micro-LEDs Using a Blister-Type Dynamic Release Polymer
Source: ACS Appl Mater Interfaces. 2025 Apr 29;17(19):28622–31. doi: 10.1021/acsami.5c01531 (PMC12086838; doi:10.1021/acsami.5c01531)
Supplement: Supplementary file 1 — am5c01531_si_001.pdf [file am5c01531_si_001.pdf]

Supporting Information

High-yield and high-accuracy mass transfer of full-color Micro-LED using a blister-type dynamic release polymer

Xinrui Huang[a,b], Qian Liu[a,b], Jinkun Jiang[a,b], Xuehuang Tang[a,b], Xin Lin[a,b], Yujie Xie[a,b], Taifu Lang[a,b], Zhonghang Huang[b], Qun Yan[b,a,c], Chang Lin[b*], and Jie Sun[b,a,d*]

[a] National and Local United Engineering Laboratory of Flat Panel Display Technology, College of Physics and Information Engineering, Fuzhou University, Fuzhou 350100, China

[b] Fujian Science & Technology Innovation Laboratory for Optoelectronic Information of China, Fuzhou 350100, China

[c] Rich Sense Electronics Technology Co., Ltd., Quanzhou 362200, China

[d] Quantum Device Physics Laboratory, Department of Microtechnology and Nanoscience, Chalmers University of Technology, Gothenburg 41296, Sweden

*Email: linchang@fjoel.cn and jie.sun@fzu.edu.cn

Table S1. Chromaticity Coordinates and the Calculation of the Color Gamut Area and Coverage.

	Coordinate (Red)	Coordinate (Green)	Coordinate (Blue)	Area (Formula 1)	Coverage (Formula 2)	Ref.
Full-color Micro-LED display	(0.683, 0.290)	(0.190, 0.742)	(0.130, 0.061)	0.1814	—	This work
NTSC	(0.670, 0.330)	(0.210, 0.710)	(0.140, 0.080)	0.1582	114.6%	1
DCI-P3	(0.680, 0.320)	(0.265, 0.690)	(0.150, 0.060)	0.1520	119.3%	2

$$\mathrm{Area} = \frac{1}{2}\left|x_R(y_G - y_B) + x_G(y_B - y_R) + x_B(y_R - y_G)\right| \qquad (1)$$

$$\mathrm{Coverage} = \frac{\mathrm{Area_{display}}}{\mathrm{Area_{standard}}} \times 100\% \qquad (2)$$

Section S1. The Structures of R/G/B Micro-LEDs.

The R/G/B Micro-LEDs used in this work (Figure S1) are all based on flip-chip structures, which offer enhanced heat dissipation and higher luminescence efficiency compared to vertical or front-loaded structures. The epitaxial layer material for blue and green Micro-LEDs is GaN, while the epitaxial layer material for red Micro-LEDs is AlGaInP. Subsequently, the R/G/B COWs are obtained through the chip fabrication processes such as photolithography, etching, metal deposition, and chemical-mechanical polishing. ITO has high conductivity and good transparency, which helps to increase the external quantum efficiency as well as improve the electrode contact performance.[3] SiO_2 is used as a passivation layer to cover the surface of Micro-LEDs, preventing the external environment, such as moisture and oxygen, from damaging the device.[4]

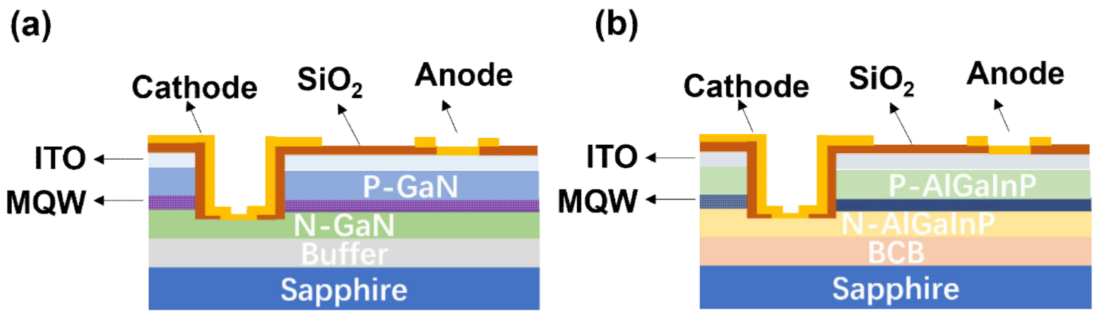

Figure S1. (a) Schematic structure of blue and green Micro-LEDs. (b) Schematic structure of red Micro-LED.

Section S2. Optoelectronic Characteristics of the R/G/B Micro-LEDs on COWs.

The EL spectra exhibit distinct emission peaks at 648.2 nm, 514.4 nm, and 456.6 nm for the red, green, and blue Micro-LEDs, respectively, with full-width at half-maximum (FWHM) values of 31.6 nm, 26.6 nm, and 20.1 nm. Narrower FWHM values are usually associated

with higher color purity and luminous efficiency.

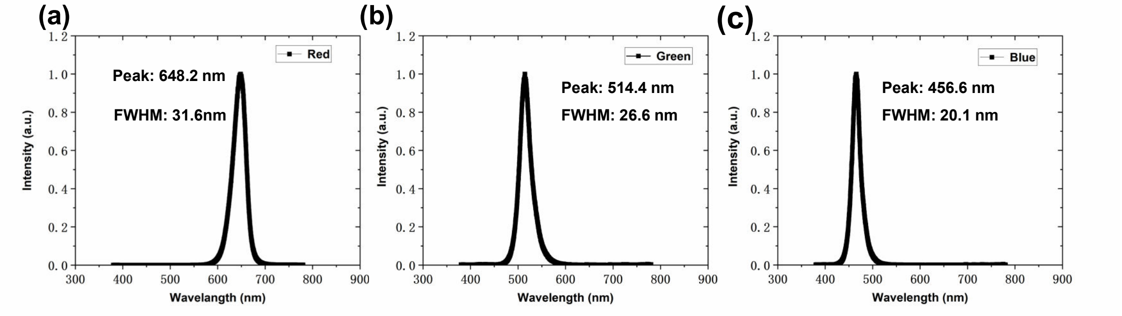

Figure S2. (a-c) The EL spectra of the R/G/B Micro-LEDs on COWs, respectively.

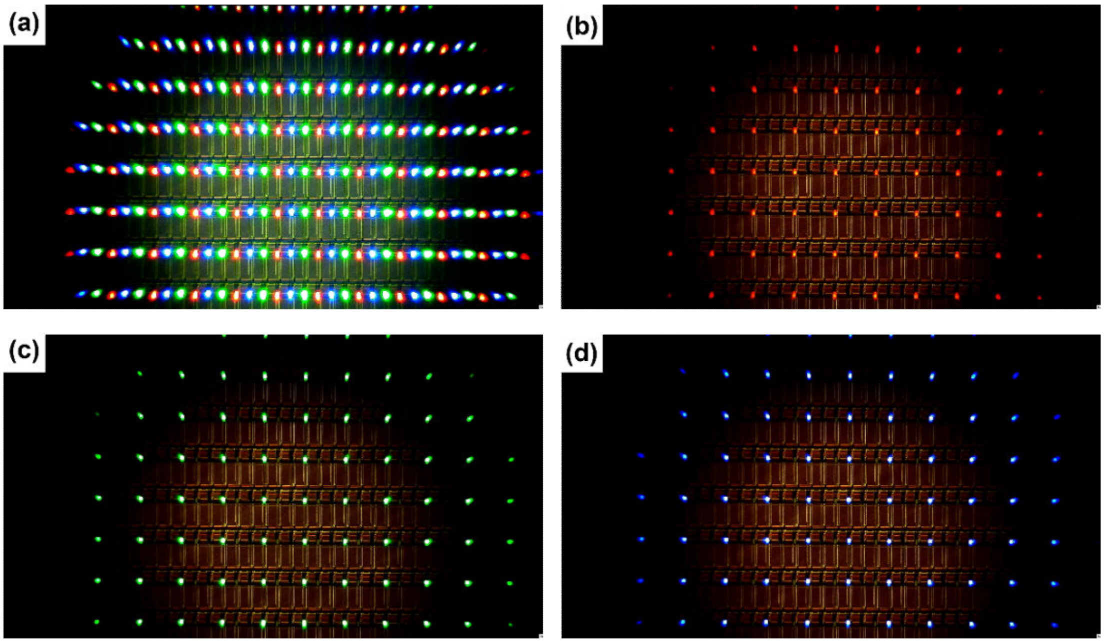

Figure S3. (a-d) The large-area lighting of a full-color Micro-LED display with red, green and blue.

References

(1) Lien, A.; Kang, C. T.; Zheng, W. W.; Chen, L. X.; Chen, Y. H.; Program, S. b. G. I. R. T. Novel Color Gamut Area Specification. In SID Symposium Digest of Technical Papers, **2014**; Wiley Online Library: Vol. 45, pp 1255-1258.

(2) Ryu, B.; Kim, K.; Ha, Y.; Bae, J.; Lee, S.; Song, J.; Lee, K.; Lee, J.; Kim, K.; Kim, H. New RGB Primary for Various Multimedia Systems. Journal of Information Display **2014**, 15 (2), 65-70.

(3) Zhou, S.; Cao, B.; Liu, S.; Ding, H. Improved Light Extraction Efficiency of GaN-based LEDs with Patterned Sapphire Substrate and Patterned ITO. Optics & Laser Technology **2012**, 44 (7), 2302-2305.

(4) Liu, Z.; Lu, Y.; Cao, H.; Maciel Garcia, G. I.; Liu, T.; Tang, X.; Xiao, N.; Aguileta

Vazquez, R.; Nong, M.; Li, X. Etching-free Pixel Definition in InGaN Green Micro-LEDs. Light: Science & Applications **2024**, 13 (1), 117.
